# The Fifth Domain of Beta 2 Glycoprotein I Protects from Natural IgM Mediated Cardiac Ischaemia Reperfusion Injury

**DOI:** 10.1371/journal.pone.0152681

**Published:** 2016-03-31

**Authors:** Peng Zhang, James C. Weaver, Gang Chen, Julia Beretov, Tatsuya Atsumi, Miao Qi, Ravinay Bhindi, Jian C. Qi, Michele C. Madigan, Bill Giannakopoulos, Steven A. Krilis

**Affiliations:** 1 Department of Infectious Diseases, Immunology and Sexual Health, St George Hospital, Sydney, Australia; 2 Department of Medicine, University of New South Wales, Sydney, Australia; 3 Department of Cardiothoracic Surgery, Tianjin Medical University General Hospital, Tianjin, China; 4 Department of Cardiology, St George Hospital, Sydney, Australia; 5 Anatomical Pathology, SEALS St George Hospital, Sydney, Australia; 6 Division of Rheumatology, Endocrinology and Nephrology, Hokkaido University Graduate School of Medicine, Sapporo, Japan; 7 Department of Cardiology, Royal North Shore Hospital, Sydney, Australia; 8 Save Sight Institute, University of Sydney and Sydney Eye Hospital, Sydney, Australia; 9 School of Optometry and Vision Science, University of New South Wales, Sydney, Australia; 10 Department of Rheumatology, St George Hospital, Sydney, Australia; Ehime University Graduate School of Medicine, JAPAN

## Abstract

Reperfusion after a period of ischemia results in reperfusion injury (IRI) which involves activation of the inflammatory cascade. In cardiac IRI, IgM natural antibodies (NAb) play a prominent role through binding to altered neoepitopes expressed on damaged cells. Beta 2 Glycoprotein I (β2GPI) is a plasma protein that binds to neoepitopes on damaged cells including anionic phospholipids through its highly conserved Domain V. Domain I of β2GPI binds circulating IgM NAbs and may provide a link between the innate immune system, IgM NAb binding and cardiac IRI. This study was undertaken to investigate the role of Β2GPI and its Domain V in cardiac IRI using wild-type (WT), Rag-1 ^-/-^ and β2GPI deficient mice. Compared with control, treatment with Domain V prior to cardiac IRI prevented binding of endogenous β2GPI to post-ischemic myocardium and resulted in smaller myocardial infarction size in both WT and β2GPI deficient mice. Domain V treatment in WT mice also resulted in less neutrophil infiltration, less apoptosis and improved ejection fraction at 24 h. Rag-1 -/- antibody deficient mice reconstituted with IgM NAbs confirmed that Domain V prevented IgM NAb induced cardiac IRI. Domain V remained equally effective when delivered at the time of reperfusion which has therapeutic clinical relevance.Based upon this study Domain V may function as a universal inhibitor of IgM NAb binding in the setting of cardiac IRI, which offers promise as a new therapeutic strategy in the treatment of cardiac IRI.

## Introduction

The World Health Organization has estimated that 48% of all deaths due to non-communicable disease in 2008 (17 million deaths worldwide) resulted from cardiovascular disease.[1] A significant proportion of these deaths are due to acute myocardial infarction as a consequence of atherothrombotic coronary artery occlusion. Prognosis after acute myocardial infarction is primarily dependent upon the amount of myocardium that is subjected to irreversible injury.[2–4] Timely reperfusion is the gold standard treatment, however restoration of coronary flow and re-oxygenation is associated with an exacerbation of tissue injury termed ‘ischemia reperfusion injury’ (IRI).[5] It is recognized that IRI may contribute up to 50% of final infarct size during acute coronary occlusion with reperfusion.[5]

An important aspect of cardiac IRI is an inflammatory response. This begins in the peri-reperfusion period and may continue for the ensuing hours and days.[6] A self-perpetuating cycle of ongoing activation is driven by chemokine signaling, cytokine release, complement activation, release of reactive oxygen species and neutrophil infiltration.[5,7,8] The earliest inflammatory pathway to be activated involves the innate immune system.[9] Even though cardiac IRI is a sterile process, this inflammatory response has many similarities to that seen in microbial ligand-pattern recognition receptor interactions during infections. In the case of IRI the analogous ligands are termed “damage-associated molecular patterns”.[10,11] Ischemic damage to endothelial cells results in changes in surface molecule expression of neoantigens that are the target of naturally occurring IgM antibodies (NAbs). Nonmuscle myosin heavy chain II is one such neoantigen exposed during IRI in intestinal,[12,13] skeletal[12] and myocardial[14] murine models. A number of other relevant neoantigens have been identified and include phosphatidyserine[15] and oxidized phophatidylcholine.[16]

Natural antibodies are germline encoded and produced primarily by B1 B lymphocytes in the absence of external antigen stimulation.[17] Despite low affinity and restricted epitope specificities they bind to these altered and exposed neoantigens,[14,16–18] which results in complement activation, through the lectin pathway.[19] Evidence supporting NAbs amplification of inflammatory tissue injury is derived from studies in antibody deficient recombination activation gene-deficient (Rag-1 ^-/-^) mice. These mice are protected from IRI with injury restored through reconstitution by IgM obtained from normal mouse sera.[20–22] Based upon these concepts the current paradigm of cardiac IRI comprises intrinsic cellular ischemic injury and an extrinsic inflammatory response initiated by the innate immune system and NAbs.

Beta 2 Glycoprotein I (β2GPI) is an abundant 43kDa circulating plasma protein[23] that plays an important role in vascular biology and may provide another link between the innate immune system and tissue injury during IRI. It is highly conserved across species suggesting it has important functions.[24] It is an integral part of the innate immune system and plays a physiological role that includes binding to damaged endothelial anionic phospholipids[25] and the binding to, and clearance of, apoptotic cells.[26] β2GPI consists of 5 domains (domains I-V); Domain V is unique and contains the anionic phospholipid binding site that is identical in all mammals.[24] In contrast Domain I contains the antibody binding site [27,28] which is cryptic due to the fact that β2GPI likely circulates in a circular configuration in-vivo with Domain I interacting with Domain V. Domain I is exposed only after Domain V binds to its ligand(s) on damaged cell surfaces.[29] Domain I is also exposed when β2GPI binds Streptococcus Pyogenes through the conserved Domain V.[30] In this latter context exposure of Domain I on the Streptococcus surface may allow Nabs to bind Domain I leading to an effective immune response against the pathogen.

The role of β2GPI in cardiac IRI has not been explored to date, however, its relevance is supported by a previous human autopsy study. In patients who died within 14 days of an acute myocardial infarction there was evidence of endogenous β2GPI deposition within the area of cardiac ischemia.[31] β2GPI deposition was not seen in non-ischemic myocardial samples from patients who died from other causes.

Our study was designed to assess whether endogenous β2GPI has a role in modulating cardiac IRI. As Domain V is highly conserved and has been demonstrated to bind various neoepitopes[32–34] which are also bound by natural IgM antibodies[15,16] we wanted to assess its therapeutic potential in cardiac IRI independent of β2GPI Domains I-IV. This was deemed necessary in view of the confounding effect of having Domain I available for NAb binding, which can initiate an immune response in its own right.

## Methods

### Animals

Mice for cardiac IRI experiments were between 11 and 15 weeks of age. C57BL/6 wild type (WT) and Rag-1 ^-/-^ mice on a C57BL/6 background were purchased from the Biological Resources Facility (Perth, Western Australia). β2GPI deficient mice (β2GPI ^-/-^) on a C57BL/6 background were bred as previously described.[35] Mice were kept under pathogen free conditions and the institutional animal ethics committee approved all experiments.

### Cardiac IRI method

A well-established model of left anterior descending (LAD) coronary artery ligation was used as described previously.[36]

The mouse was initially anaesthetized with 2% isoflurane, shaved and placed on a board with paws taped for electrocardiographically gated echocardiography. The mice were then further anaesthetized with ketamine (80 mg/kg) and xylazine (10 mg/kg) prior to endotracheal intubation. Local anaesthetic was infiltrated into the wound site perioperatively, 0.25% bupivacaine. Buprenorphine (0.1 mg/kg, subcutaneously) was administered following intubation for analgesia. Ventilation was maintained with a Hugo Sachs Minivent 845 (Harvard Apparatus, Holliston, MA) with stroke volume 200 μl at 125 strokes per min. The body temperature was maintained at 37 ± 0.5°C by using a heating pad and monitored with a rectal probe. After anaesthesia induction to confirm adequate anaesthesia the withdrawal reflex, general body tone, the colour of extremities, heart rate and respiratory rate were assessed. Using a Leica M320 surgical microscope (Leica Microsystems GmbH, Wetzlar, Germany) a thoracotomy was performed by opening the left 4th intercostal space with the incision exposing the heart after spreading the ribs using a self-retaining retractor. The pericardium was opened and the proximal LAD coronary artery snared using a 7/0 suture, 1 mm distal to the left atrial appendage. The suture was tied over a short segment of polyethylene tubing (PE 10), predominantly to anchor and stabilize the PE tubing in place. A second suture was tied immediately distal to the first suture with vessel occlusion confirmed by visualization and digital video recording of myocardial pallor. Ischemia was maintained for 30 min, following which the sutures were released and the chest closed with layered sutures. An 18-gauge cannula was used as a chest tube to create negative intra-thoracic pressure to assist in lung reinflation prior to extubation. In the sham procedure group, a suture was only passed underneath the LAD coronary artery but not tightened.

### Assessment of infarction size

Infarction size was assessed at 24 h after reperfusion, either by histology and/or troponin I. For infarction size by histology the mice were again anaesthetized, they undergo echocardiography and were then intubated and ventilated. The LAD was re-ligated at the same site as the previous day and 0.3 ml 1% Evans Blue injected into the left ventricle for measurement of the “Area at Risk” (AAR).

Mice underwent euthanasia with 0.25ml Pentobarbital delivered into the left ventricular cavity. The heart was then excised and 1 mm serial sections cut using mouse Heart Matrix (Roboz Surgical Instrument, Gaithersburg, MD). These were then stained with 1% triphenyl-2, 3, 4- tetrazolium-chloride (TTC) for 20 min at 37°C. Finally the sections were photographed on both sides using a Canon EOS 600D camera with Canon EF 100 mm lens and Macro ring lite MR-14Ex light source (Canon Inc, Ota, Tokyo, Japan). On these images the AAR and infarct size were quantified using ImageJ software (NIH, Bethesda, MD). The left ventricular volume was measured from the first slice above the suture site down to the apex, excluding the papillary muscles. Both sides of a 1 mm section of myocardium were quantitated and the average of both sides calculated. The non-AAR was defined as the myocardium that stained blue with Evans Blue and this was quantitated by planimetry. Subsequently the AAR was calculated (total myocardium—non AAR) and then the infarction size quantitated by planimetry as a percentage of the AAR. The individual performing the surgery and the two investigators quantifying infarct size, tunnel assay, neutrophil infiltration and troponin I levels were blinded to treatment allocation and mouse strains.

Infarction size was also assessed after 24 h reperfusion using an ultrasensitive murine troponin I ELISA (Life Diagnostics, West Chester, PA).[14] Blood was collected from the inferior vena cava using a 29 gauge needle immediately prior to euthanasia. Serum was stored at -20°C and all samples were analyzed at the same time as specified in the manufacturer’s instructions.

Mice were excluded only if the surgeon confirmed failure of surgery to induce ischemia or the investigator analyzing the TTC stains confirmed failure to demonstrate myocardial necrosis. Both investigators were required to concur on exclusion whilst remaining blinded to treatment allocation. No mice were excluded from the troponin I experiments.

### Immunohistochemistry

A separate group of animals were used for immunohistochemistry staining (IHC). For assessment of neutrophil infiltration and β2GPI deposition, myocardial tissue was fixed in Zinc (BD Biosciences, San Jose, CA) for 24 h, washed in 70% ethanol, processed and embedded in paraffin blocks. For assessment of apoptosis, myocardial tissue was fixed in 10% neutral buffered formalin (Sigma-Aldrich, St. Louis, MO) for 24 h and also embedded in paraffin. Four μm sections were cut from the paraffin blocks. Staining was performed on the four μm tissue sections cut from the paraffin blocks.

For neutrophil infiltration a primary rat anti-Gr-1 antibody (BD Biosciences) was diluted 1:50 and visualized by a biotin-conjugated anti–rat Ig, Streptavidin-linked HRP complex and 3,3’-diaminobenzidine as substrate. The Gr-1 positive cells represent both neutrophils and inflammatory Ly6C monocytes, however, at the 24 h time point predominantly reflect neutrophils.[37,38] Myocardial samples were mounted 2 per slide and scanned using an Aperio Scanscope XT digital scanner (Leica Biosystems GmbH, Nussloch, Germany). Gr-1 positive cells were then quantified by counting the number of immunoreactive cells with Aperio eSlide Manager software (Leica Biosystems) in 6 myocardial slices (each slice separated by >200μm) per mouse. The total number of cells over the 6 sections were recorded for each mouse.

Apoptotic cells were quantitated using the Deadend Fluorometric TUNEL system (Promega, Madison, WI). From the formalin fixed blocks, two sections from within the AAR, one from the base and one from the apex, were mounted on a single slide. In brief, the tissue is deparaffinized, rehydrated using graded alcohol washes and fixed in 4% formaldehyde. The tissue is then treated with recombinant Terminal Deoxynucleotidyl Transferase enzyme that forms a polymeric tail on apoptotic cells. This can then be incorporated by fluorescein-12-dUTP at 3′-OH DNA ends and the fluorescein visualized using a Zeiss AxioVert A1 light microscope (Carl Zeiss, Jena, Germany). Total number of apoptotic nuclei over the two segments was recorded per mouse.

β2GPI staining was performed on the paraffin sections after rehydration and antigen retrieval using a 95°C water bath and 0.01M Citrate buffer (pH 6.0). Sections were incubated overnight at 4°C with an anti-rabbit β2GPI (1:250 dilution) primary antibody, and finally incubated for an additional 2 h in secondary antibody, donkey anti-rabbit Alexa 488 (Invitrogen, Carlsbad, CA). Two sections from within the AAR, one from the base and one from the apex, were mounted on a single slide and analyzed using a Zeiss AxioVert A1 light microscope (Carl Zeiss) and Zeiss LSM700 scanning laser confocal microscope with image software (Zen 2011, Carl Zeiss MicroImaging). Two observers blinded to treatment allocation independently scored β2GPI deposition as; 0- no staining, 1- minimal staining involving one section, 2-mild staining involving both segments and not transmural, 3-moderate staining (intermediate between scores 2 and 4), 4-severe staining involving the majority of the AAR and transmural in extent. Disagreement between observers was resolved by consensus.

### Enzyme-linked immunoabsorbent assay to detect β2GPI in serum

Blood was collected from the inferior vena cava 24h after IRI. Serum was stored at -80°C prior to analysis as previously described.[39] In summary, samples were diluted 100-fold and added to a 96-well plate coated with 100 microlitres of β2GPI at 10 micrograms per ml in coating buffer. A rabbit polyclonal anti-β2GPI antibody (100nM) directed against Domain I of murine β2GPI was used as the primary antibody and incubated for 1 hour at room temperature. Then anti-rabbit immunoglobulin conjugated to alkaline phosphatase was incubated for an additional hour as the secondary antibody (1:1500 dilution). Samples were read using the appropriate chromogenic substrate.

### Treatments

The respective treatments were delivered by intracardiac injection (directly into left ventricular cavity for TTC studies in WT and β2GPI^-/-^ mice and neutrophil studies) either; 5 min prior to IRI, or after ischemia but immediately prior to reperfusion. For the dose escalation experiments, apoptosis on IHC and experiments in Rag-1 ^-/-^ mice the therapies were delivered intravenously through tail vein injection. Recombinant human Domain V (rhDomain V) 40 μmol/L (60 μg/100 μL) and rhβ2GPI 40 μmol/L (200 μg/100 μL) or recombinant murine β2GPI (rmβ2GPI) 40 μmol/L were administered.[27] Assuming a blood volume of 1.9 ml in the mice studied this results in a circulating concentration of 2 μmol/L; equivalent to the murine physiological concentration of β2GPI. Controls consisted of either normal saline 100 μl or no treatment. Sham mice were treated with normal saline. The murine peptide 9 sequence (SSYTVEAHS) and control peptide (AQCMPDVRIQTA) were as previously described.[40] Both were diluted in 100 μL of normal saline to make a final concentration of 40 μmol/L. For experiments in Rag-1 ^-/-^ mice the treatments were delivered at 2 time points. Prior to IRI the mice were treated with either normal saline or 400 μg pooled murine IgM[21] (CD Creative Diagnostics, Shirley, NY). After 30 min ischemia and prior to reperfusion they were treated with either normal saline control or rhDomain V. Randomization, blinding and tissue analysis was conducted in accordance with the recently published consortium statement.[41]

### Echocardiography

Transthoracic 2D echocardiographic studies were performed with the mice under isoflurane anesthesia prior to and 24 h after IRI. A Vevo 770 system (Visualsonics Inc, Toronto, Canada) equipped with a dedicated 30-MHz small animal transducer was used to obtain images that were then analyzed in an offline workstation.[42] In the long axis images, planimetry of LV dimensions from end-diastole to end-systole allowed calculation of the LV ejection fraction.

### Statistics

Data are presented as mean ± SEM. Comparisons between two groups were performed using a Student’s t-test. Comparison between three or more groups was performed using an ANOVA. For neutrophil and TUNEL IHC samples the sum of total stained nuclei of all the samples within one mouse was taken. Correlation between troponin I levels and infarction size on TTC was assessed using the Pearson correlation co-efficient. Analysis was performed using SPSS version 22 (SPSS Inc. Chicago, IL). Differences were considered statistically significant at p < 0.05.

### Study Approval

All animal studies were approved by the University of New South Wales Animal Care and Ethics Committee (ACEC) approval number 13/144B. Total number of mice used for the entire study was 328.

## Results

### β2GPI localizes to the area at risk after IRI

β2GPI was demonstrated to be present within the myocardium of WT mice subjected to 30 min ischemia and 24 h reperfusion. β2GPI deposition occurred within the AAR in control WT mice treated with normal saline, no treatment and rhβ2GPI (S1 Fig). It was not present outside the ischemic AAR and almost undetectable in sham LAD ligation.

### Domain V protects from cardiac IRI

**Fig 1:**
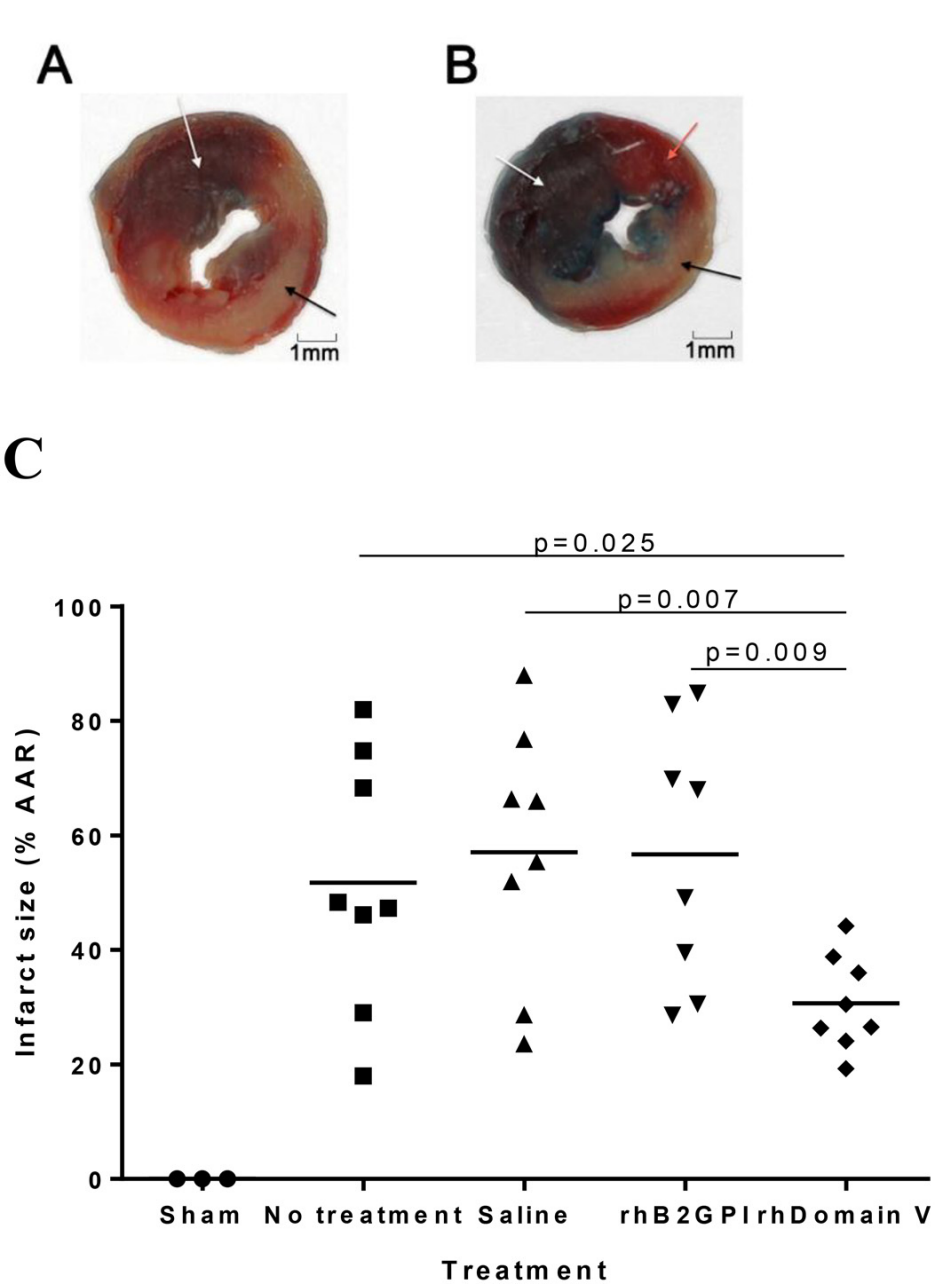
Infarction size in C57BL/6 WT mice. (A) Representative images of myocardial infarction in a mouse treated with control after 30 min ischemia and 24 h reperfusion. Section of myocardium at the mid papillary level stained with Evans Blue and 1% triphenyl-2,3,4- tetrazolium-chloride for 20 min at 37°C. The area stained blue with Evans Blue represents the non AAR (white arrow). The remaining area consists of myocardial infarction staining pale (black arrow) and the surrounding AAR that did not sustain myocardial necrosis. (B) Representative image of myocardial infarction in a mouse treated with rhDomain V. The pale staining area of infarction (black arrow) is significantly smaller than control. Consequently the extent of salvaged myocardium (orange arrow) is significantly larger. Sections photographed using Canon EOS 600D camera with Canon EF 100 mm lens and Macro ring lite MR-14Ex light source. (C) Points representing myocardial infarction size defined by 1% TTC staining. Mice treated with sham LAD occlusion demonstrated no myocardial infarction (n = 3). Control groups treated with normal saline 100μL, no treatment or human β2GPI all had similar infarction size. Mice treated with rhDomain V had a significant reduction in infarction size (n = 8 per group). AAR; Area at Risk. Individual points represent infarct size as a % of AAR of a single mouse. The horizontal line for each study group represents the mean.

To investigate whether rhDomain V protected from cardiac IRI, the protein or control was administered prior to IRI in WT mice. Domain V 40 μM given prior to IRI reduced infarction size by 44% compared with control treated animals (Fig 1). The rhβ2GPI did not protect from IRI with infarct size similar to that seen in controls (56.7% ± 8%, n = 8, p = NS). There was no difference in the AAR between the treatment groups (Normal saline 34.7 ± 2.5%, No treatment 31.7 ± 1.7%, rhDomain V 37.3 ± 2.1%, rhβ2GPI 41.7 ± 3.4%, n = 8, p = NS). During surgery or within the 24 h reperfusion period there were 5 deaths (13.8%); 1 mouse treated with normal saline, 2 mice treated with rhΒ2GPI and 2 with no treatment. There were no deaths in WT mice treated with rhDomain V.

The reduction in infarction size resulted in a preservation of ejection fraction on echocardiography in the rhDomain V treated WT mice. Ejection fraction was higher in the rhDomain V treated mice (36.4 ± 2.0%, n = 15) compared with controls (26.8 ± 3.2%, n = 24, p = 0.034). Sham mice had an ejection fraction of 53.2 ± 3.8% (n = 3).

### Domain V reduces Gr-1 positive cell infiltration

**Fig 2:**
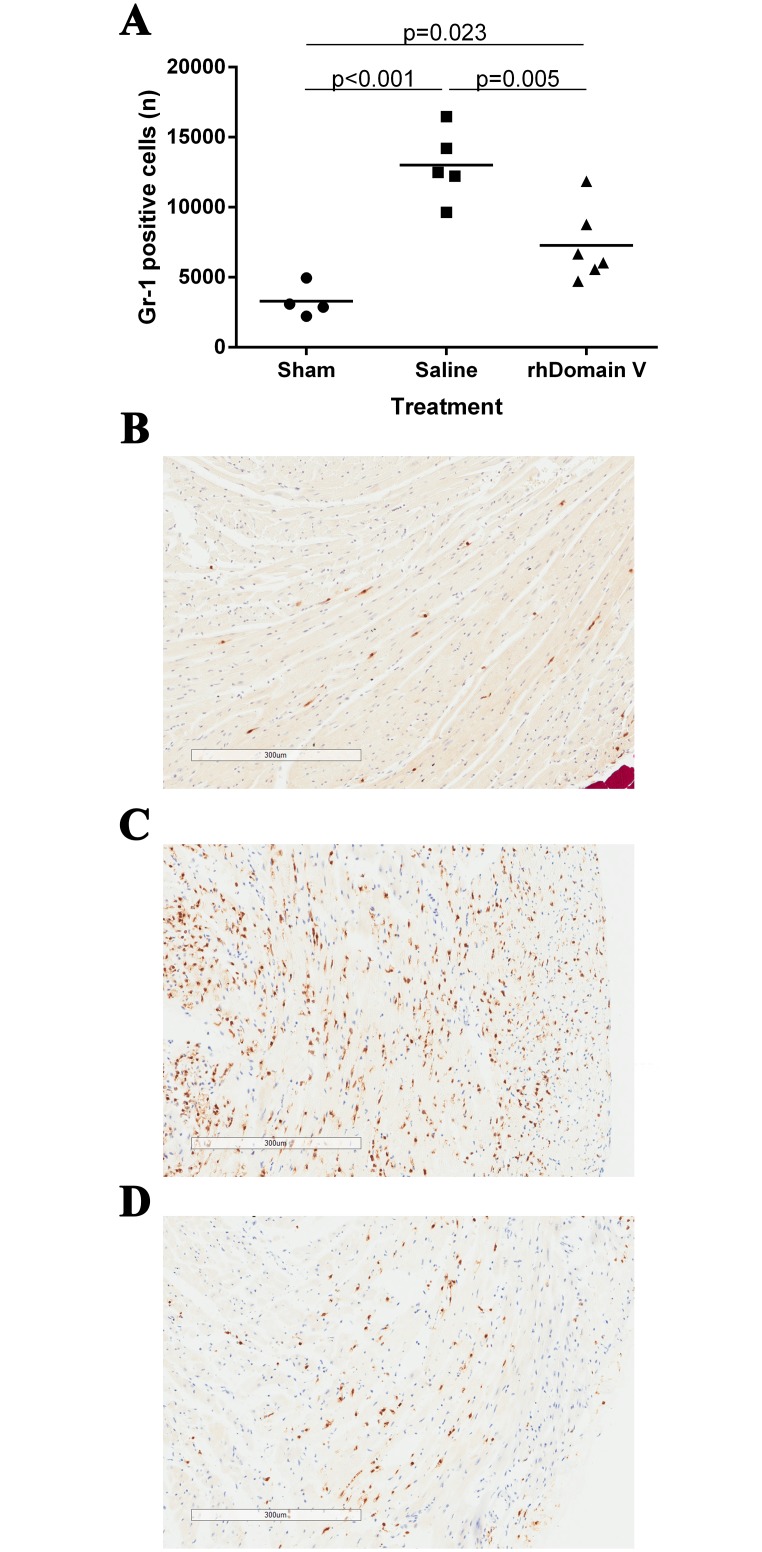
Anti Gr-1 staining. (A) Treatment with rhDomain V prior to IRI resulted in less Gr-1 positive cell infiltration compared with control. (B) Representative images from within the area of risk showing immunolabelling for anti-Gr-1 in a mouse treated with sham LAD occlusion, (C) control and (D) rhDomain V. Images obtained using Aperio Scanscope XT digital scanner at 10x magnification.

Using the anti Gr-1 antibody on paraffin embedded sections immunoreactive cells were localized to the AAR in WT mice subjected to IRI. Small numbers of Gr-1 positive cells were present in sham LAD ligation. Domain V treatment prior to IRI resulted in lower levels of Gr-1 positive cells 24 h after reperfusion compared with mice treated with normal saline (Fig 2, n = 6).

### Domain V remains effective at a clinically meaningful time point

In clinical practice therapies for IRI can only be delivered after ischemia but prior to reperfusion. Domain V was also delivered after cardiac ischemia and immediately prior to reperfusion. It remained effective at protecting from IRI with infarction size similar to that when the protein was delivered prior to IRI (32.2 ± 5.8 vs. 30.7 ± 2.9%, p = 0.82, n = 8 per group). There remained a significant 43.5% reduction in infarction size when rhDomain V was compared with normal saline (32.2 ± 5.8 vs. 57.1 ± 7.9%, p = 0.023).

### Dose escalation studies and effect on apoptosis

**Fig 3:**
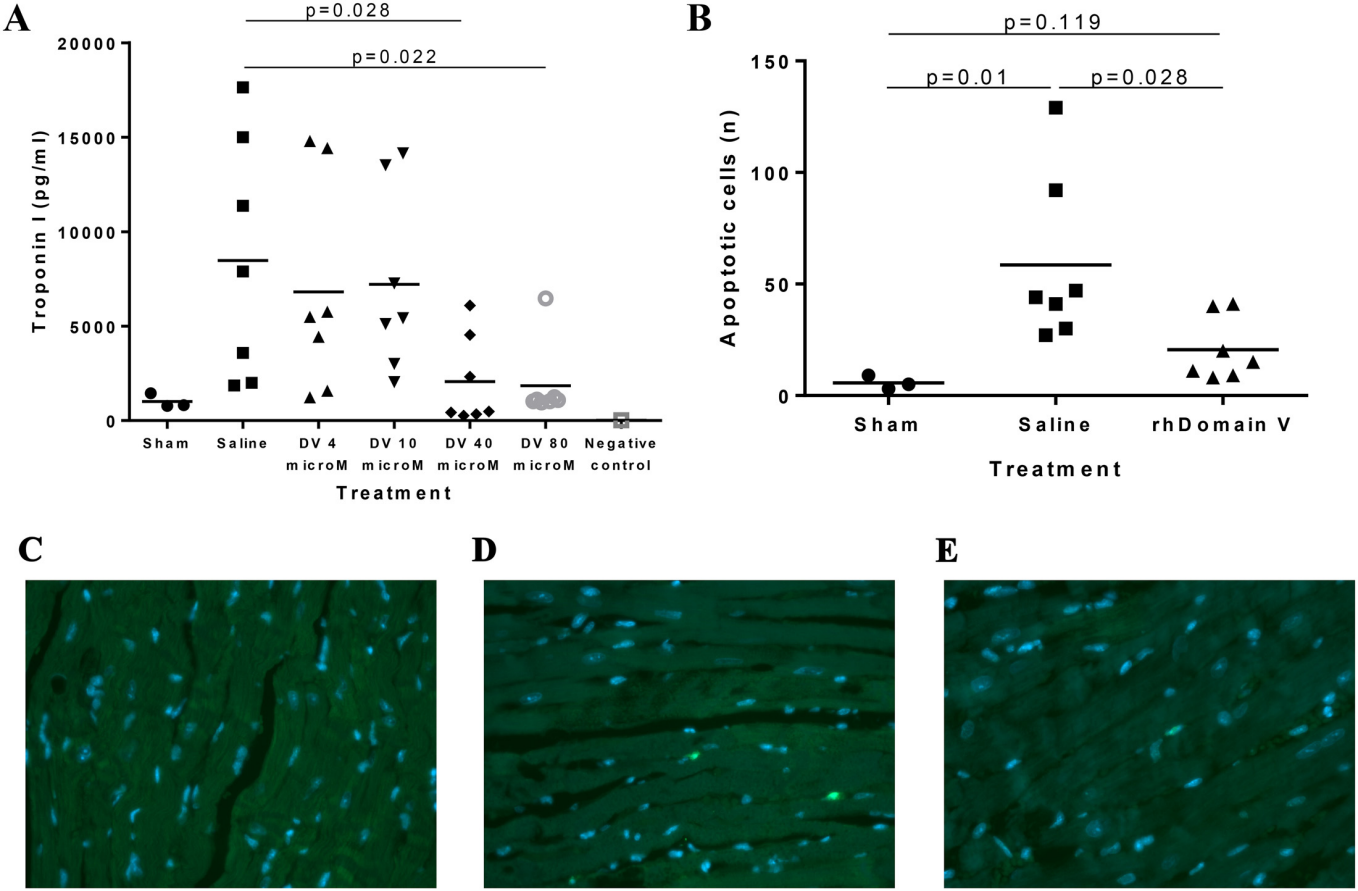
Optimal dose of Domain V in cardiac IRI. (A) C57BL/6 WT mice were treated with control or escalating doses of rhDomain V (n = 7) and assayed for troponin I by ELISA. (B) Mice treated with rhDomain V 40 μM (n = 7) had lower levels of apoptosis compared with control (n = 7). (C) Representative image from within the AAR showing apoptotic cells using the Deadend Fluorometric TUNEL system in a mouse treated with sham LAD ligation, (D) saline and (E) rh Domain V. Images obtained using a Zeiss AxioVert A1 light microscope. DV = Domain V. Points represent number of apoptotic cells in individual mouse cardiac tissue. The horizontal line for each group represents the mean.

A dose escalation study was performed in a separate group of mice to clarify the optimal dose of rhDomain V for protection from cardiac IRI. Extent of myonecrosis was defined by ultrasensitive troponin I level 24 h after IRI. Domain V or control was delivered after ischemia and prior to reperfusion. It was confirmed that an intravenous dose of 40 μmol/L rhDomain V is the optimal dose over a range of 4–80 μM (Fig 3A). The control and rhDomain V 40 μmol/L groups had cardiac tissue collected for quantitation of apoptotic cells. Domain V treatment resulted in a significant reduction in apoptotic cells compared with control (n = 7) (Fig 3B).

### Serum β2GPI and cardiac IRI

**Fig 4:**
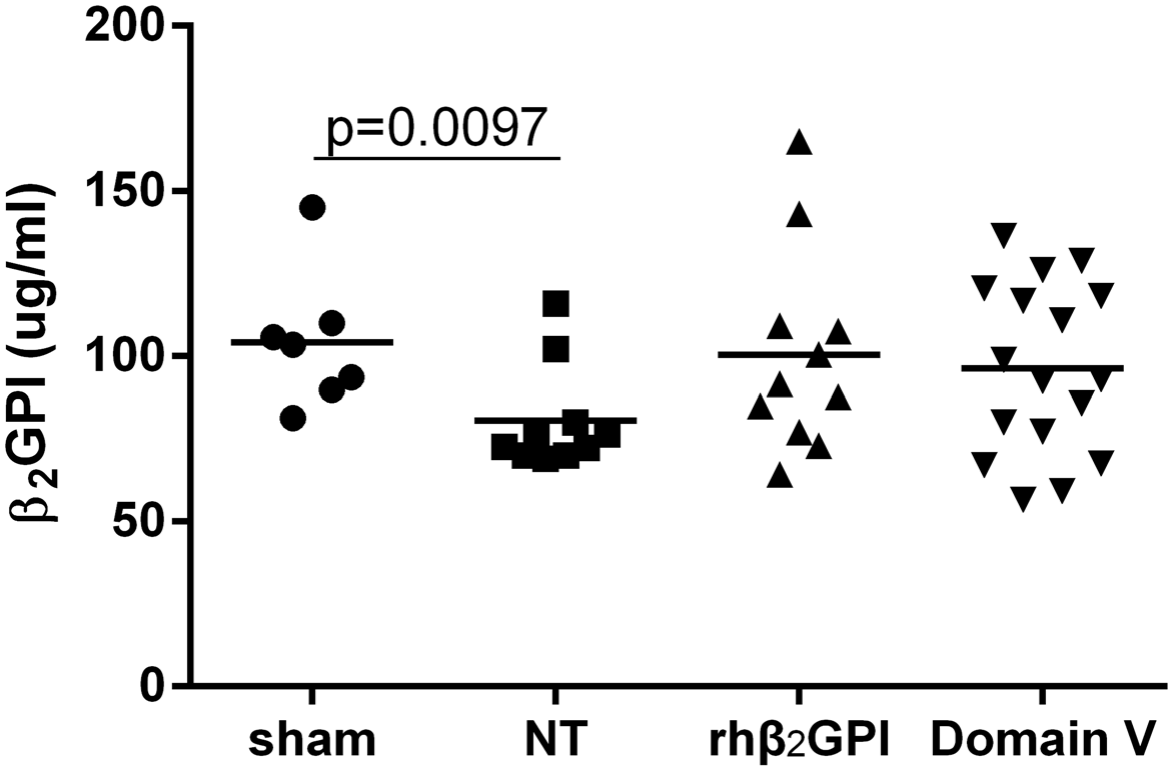
Serum β2GPI levels after cardiac IRI. Serum total β2GPI levels in mice 24 h after cardiac IRI. NT = No treatment. rhβ2GPI = recombinant human β2GPI. Domain V = recombinant human Domain V. Horizontal line for each group represents mean.

In mice undergoing sham procedure the mean serum β2GPI was 104.1 ± 21 μg/ml (n = 7) and this was significantly reduced to 80.5 ± 16 μg/ml in the control group (n = 10) 24 h after IRI (Fig 4). In the groups treated with rhDomain V (n = 17) and rhβ2GPI (n = 11) there was no fall in serum β2GPI levels which remained comparable to sham treated mice. This fall in native serum β2GPI during cardiac IRI was taken to represent consumption of circulating β2GPI due to tissue binding which was prevented by rhDomain V and rhβ2GPI.

### Domain V reduces endogenous mouse β2GPI cardiac tissue deposition

β2GPI deposition was evident within the AAR in WT mice subjected to IRI. Domain V treatment resulted in lower levels of endogenous β2GPI deposition compared with mice treated with controls (1.7 ± 0.3 vs. 2.7 ± 0.3, p = 0.04, n = 10 per group). The lower myocardial β2GPI and higher serum β2GPI in those treated with rhDomain V was taken to represent competitive and preferential binding of rhDomain V within the AAR.

### Murine Peptide 9 did not protect from cardiac IRI

Peptide 9 has previously been demonstrated to protect from murine mesenteric IRI.[40] It is a small peptide derived from murine Domain V of β2GPI that does not include the anionic phospholipid binding site. When delivered prior to IRI, Peptide 9 did not reduce myocardial infarction size when compared with control peptide (50.9 ± 8 vs. 55.5 ±12%, n = 7, p = NS). Left ventricular function was impaired in both groups as assessed by ejection fraction at 24 h after reperfusion (Control peptide, 22.6 ± 3.6 vs. Peptide 9, 20.7 ± 4.1%, p = NS).

### Endogenous β2GPI does not modulate cardiac IRI within 24 h

Cardiac IRI experiments were then performed in β2GPI ^-/-^ mice to allow comparison with WT mice. β2GPI ^-/-^ mice had similar infarction size to WT mice (p = NS). The infarction size after 30 min ischemia and 24 h reperfusion was similar in β2GPI ^-/-^ mice given no treatment (n = 6) or normal saline (n = 5) prior to IRI (49.1 ± 7 vs. 58.3 ± 12%, p = NS). Mice reconstituted with rmβ2GPI had similar infarction size to the β2GPI deficient mice (n = 6). There was no difference in the AAR between the groups of mice.

### rhDomain V protects β2GPI^-/-^ mice from cardiac IRI

**Fig 5:**
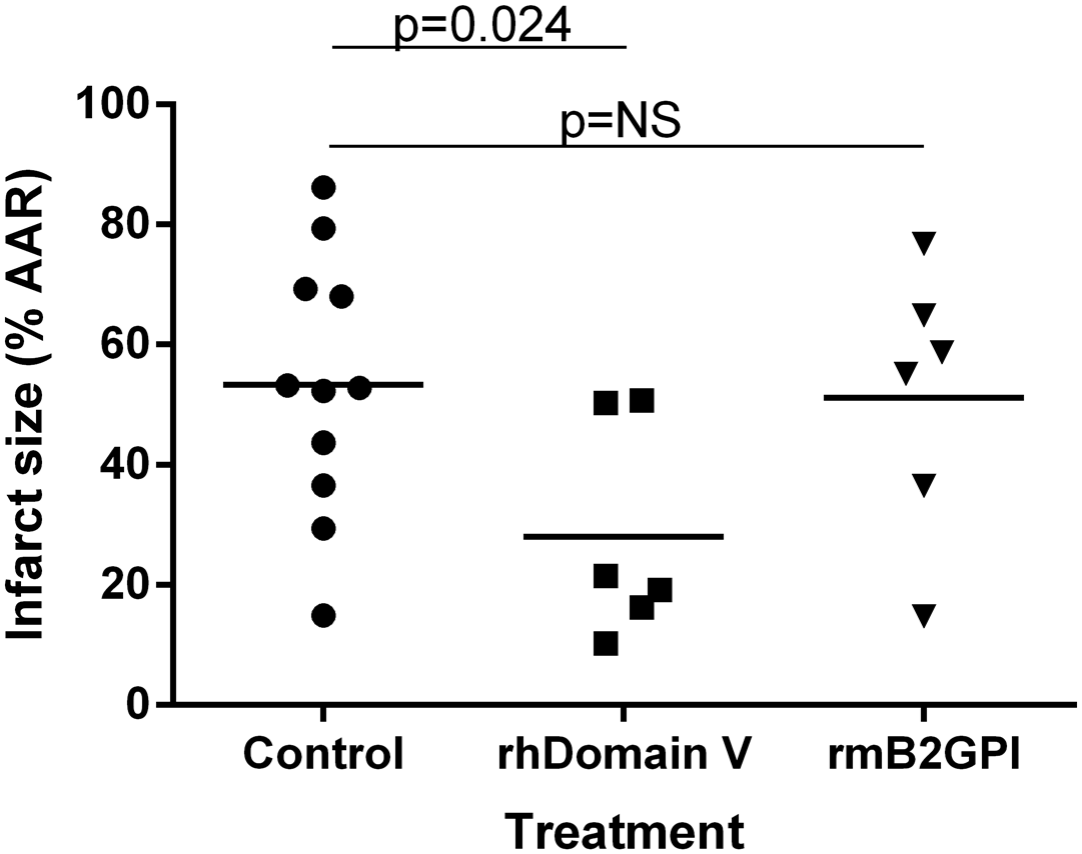
β2GPI deficient mice and cardiac IRI. β2GPI -/- mice treated with control sustained the same myocardial infarction size on 1% TTC staining as WT mice. Domain V (n = 6) protects β2GPI -/- mice from cardiac IRI compared with control (n = 11). Reconstitution with rmβ2GPI (n = 6) resulted in similar infarction size to control mice. Individual points represent infarct size as a % of AAR of a single mouse. The horizontal line for each study group represents the mean. NS = not significant.

When rhDomain V 40 μmol/L was delivered prior to IRI, the protective effect seen in WT mice was also observed in β2GPI ^-/-^ mice (Fig 5). Echocardiography after 24 h reperfusion demonstrated preserved ejection fraction in β2GPI ^-/-^ mice treated with rhDomain V (37.4 ± 6.1%, n = 6), which was similar to WT mice treated with rhDomain V.

### rhDomain V protects against cardiac IRI in Rag-1 ^-/-^ antibody deficient mice reconstituted with natural IgM

The findings in β2GPI ^-/-^ mice indicate that rhDomain V inhibits IRI through a β2GPI independent mechanism. It was hypothesized that rhDomain V may inhibit circulating NAbs binding to altered neo-epitopes during the reperfusion phase of cardiac IRI. To investigate this, experiments were performed in Rag-1 ^-/-^ antibody deficient mice. It has previously been demonstrated that Rag-1 ^-/-^ mice are protected from cardiac IRI but reconstitution with pooled mouse IgM re-establishes susceptibility to cardiac IRI. This occurred through IgM binding to an altered endothelial neoepitope.[12,13,22]

**Fig 6:**
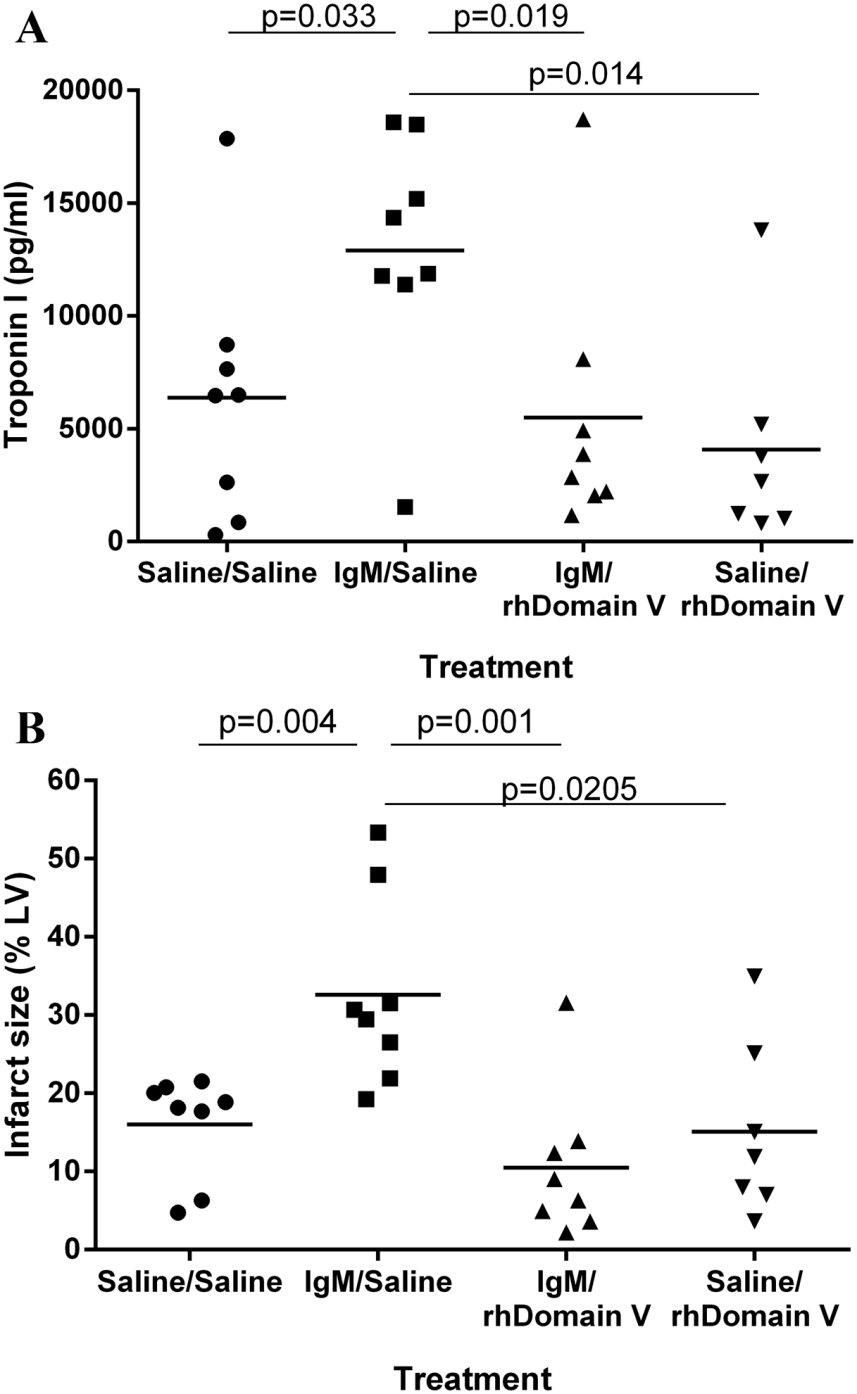
Domain V protects from cardiac IRI in Rag-1 -/- antibody deficient mice reconstituted with natural IgM antibodies. (A) Rag-1 -/- mice were treated with saline or pooled murine IgM prior to IRI. The mice treated with IgM were further treated with either saline or rhDomain V at the time of reperfusion. Domain V reduced Troponin I levels at 24 h to similar levels to the antibody deficient mice. (B) Treatment with rhDomain V reduced infarction size (as percentage of total LV) in antibody deficient mice reconstituted with pooled IgM. IgM = murine immunoglobulin M. Individual points represent infarct size as a % of LV of a single mouse. The horizontal line for each study group represents the mean.

Rag-1 ^-/-^ mice were treated with normal saline or reconstituted with pooled murine IgM (Fig 6). Reconstitution with IgM prior to IRI increased myocardial injury as defined by troponin I level at 24 h (n = 8 per group) (Fig 6A) and infarction size on TTC (Fig 6B). Domain V delivered at the time of reperfusion prevented the IgM induced myocardial injury in Rag-1 ^-/-^ mice (Fig 6A and 6B). Treatment with rhDomain V without IgM reconstitution did not further reduce infarction size. There was a good correlation between infarction size on TTC and serum troponin at 24 h (r = 0.806, p < 0.001).

## Discussion

This study confirms the hypothesis that rhDomain V protects from cardiac IRI with a 44% reduction in infarction size. These findings were reproduced in separate groups of mice demonstrating reduced neutrophil/Ly6C monocyte infiltration, apoptosis and troponin I elevation. Protection from IRI by rhDomain V is dose dependent up to physiological concentrations and remains effective when delivered at the time of reperfusion; this is analogous to the time that patients with blocked coronary arteries undergo revascularization, making it a clinically relevant therapeutic time point. Importantly, the magnitude of protection was not diluted when rhDomain V was delivered at the later time point, a finding with biological plausibility, as reperfusion is required for binding of NAbs to damaged endothelium.

It has previously been demonstrated that there is translocation of phosphatidylserine, a negative charged phospholipid to the cell surface and exposure of other neoepitopes following IRI.[43] Domain V contains the critical anionic phospholipid (phosphatidylserine) binding site of β2GPI[32,33], which also binds other IRI neoepitopes such as oxidized phophatidylcholine.[34] We speculate that the protective effect seen with rhDomain V of β2GPI is due to inhibition of natural IgM binding to cardiac IRI neoepitopes.

Previous therapies have targeted mediators of the inflammatory cascade once it has been initiated by natural antibody induced complement activation. In contrast, Domain V treatment inhibits the initiation of natural IgM mediated inflammation. Whilst there have been studies evaluating anti-inflammatory agents, on the whole there has been a failure to translate pre-clinical studies to widespread clinical use in patients. For example, two anti-inflammatory therapies showed promise in animal studies, yet failed to show benefit in patients with acute myocardial infarction. The C5 inhibitor, Pexelizumab, demonstrated a reduction in infarction size and apoptosis in animal studies[44] but then failed to improve outcomes in a large clinical study.[45] A monoclonal antibody against the leucocyte integrin CD-18 demonstrated cardioprotection in rodents by limiting neutrophil accumulation.[46,47] A subsequent study using a humanized antibody against the CD-11/CD-18 integrin receptor in 420 patients demonstrated no reduction in infarction size after primary angioplasty.[48] It has been proposed that the failure of these agents may be explained in part by their inability to inhibit C3 activation of mast cell induced injury.[14] Targeting of the inflammatory cascade very early in the pathway would provide more complete inhibition. Domain V in our study protects from IRI in a murine model of IgM reconstitution using Rag-1 ^-/-^ antibody deficient mice. In view of the protection against cardiac IRI in WT and β2GPI ^-/-^ mice when administered rhDomain V, this confirms that rhDomain V inhibits IgM NAb binding through both β2GPI dependent and independent mechanisms. Domain V also demonstrates binding to anionic phospholipids and multiple other relevant cardiac neoepitopes suggesting that it works as a universal inhibitor of NAb binding to a wider range of altered and exposed neoantigens. It is likely that the therapeutic potential of rhDomain V will be relevant for human cardiac IRI as there is complete homology between humans and mice within the phospholipid binding site.[24] Tolerability of rhDomain V in humans would require further studies. However, given that rhDomain V is not a ‘foreign’ protein and also therapeutic at physiological levels, it is likely to be well tolerated.

Peptide 9 lacks the anionic phospholipid binding site of Domain V and consequently did not protect from cardiac IRI. A previous study in mesenteric IRI demonstrated protection from reperfusion injury with Peptide 9 compared with control,[40] which highlights the mechanism of protection of β2GPI peptides is different in cardiac compared to intestinal IRI. Further emphasizing the need to assess therapies using organ specific IRI murine models.

**Fig 7:**
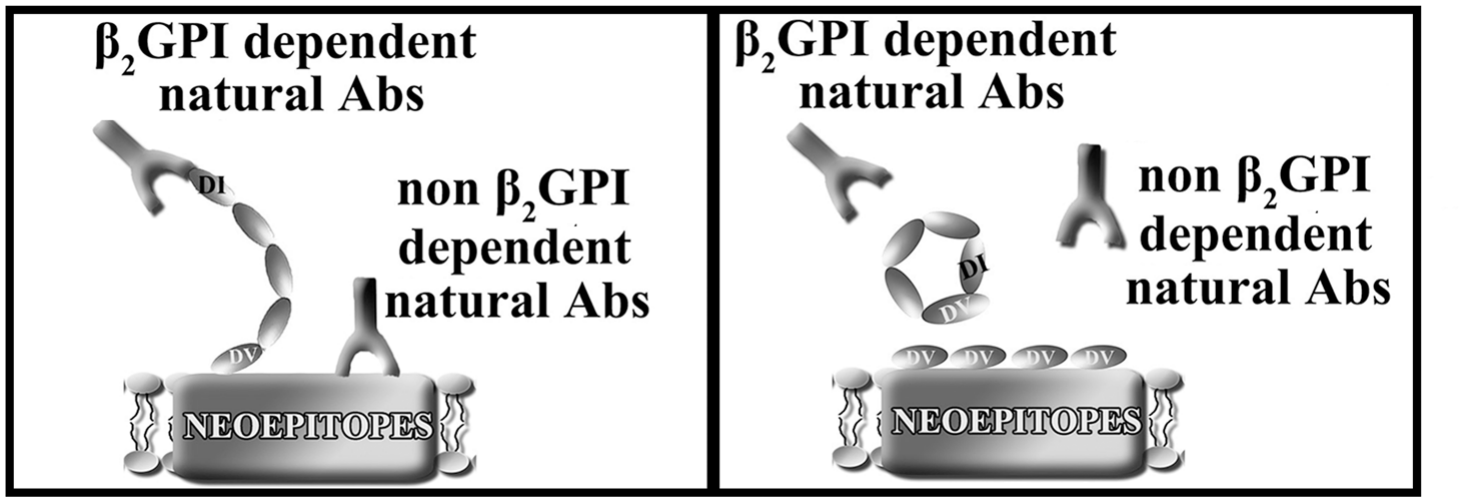
Domain V of β2GPI binds neoepitopes on cardiac ischemic tissue inhibiting IgM NAb binding. (A) Natural antibodies can bind multiple different epitopes in the setting of cardiac IRI including non-muscle myosin II and β2GPI bound to anionic phospholipids. Domain I of endogenous β2GPI is exposed upon binding of its Domain V to damaged endothelium in the setting of IRI. (B) Domain V binds neoepitopes exposed on the surface of damaged cardiac tissue. This has two effects, prevention of exposure of the Domain I cryptic epitope and binding of anti-β2GPI NAbs. It also prevents other NAbs binding to multiple non-β2GPI neoepitopes.

β2GPI deficient mice had similar infarction size to WT mice and were not protected from cardiac IRI. This finding suggests that β2GPI and anti- β2GPI NAb binding is insufficient to induce IRI alone. This concept is supported by studies in mesenteric IRI. It has been shown that reconstitution with anti-β2GPI antibodies can restore injury in Cr2 ^-/-^ mice which have a limited repertoire of natural antibodies.[49] However, reconstitution with anti-β2GPI IgG was insufficient to restore injury in Rag-1 ^-/-^ mice that are completely antibody deficient. A combination of antiphospholipid antibodies and anti- β2GPI IgG antibodies were required to restore injury in these mice.[50] In contrast, a subsequent study in Rag-1 ^-/-^ mice suggests a more pivotal role for β2GPI in mesenteric IRI. Reconstitution of antibody deficient mice with the entire antibody spectrum except anti-β2GPI antibodies (but including IgG and IgM), failed to restore tissue injury.[51] When combined with the results of the current study in β2GPI ^-/-^ mice it is proposed that β2GPI modulates cardiac IRI when present, but other NAb binding ligands are recruitable in its absence such as phosphatidylserine, oxidized PC and nonmuscle myosin heavy chain II. Taken together these studies suggest that multiple autoantibodies circulate at physiological concentrations and are responsible for inflammatory tissue injury by binding to multiple cryptic neoantigens. Domain V has the ability to prevent NAb binding to these neoantigens in both a β2GPI dependent and independent mechanism during cardiac IRI (Fig 7).

In conclusion, our study is novel since the treatment we have studied, and propose for future human studies, acts at the initiation of the natural antibody mediated inflammatory cascade in the setting of cardiac IRI. This novel therapy does not target a single neoantigen expressed by damaged heart muscle nor a single downstream inflammatory pathway but acts as a universal inhibitor of the multiple IgM Nabs that are responsible for cardiac IRI. Targeting the initiation of the inflammatory cascade may offer a therapy which may be effective in human cardiac IRI in contrast to failed therapies that focused on downstream inflammatory pathways. Prior to human studies this therapy will need to be assessed in large animal models of cardiac IRI to allow determination of long term effects, remodeling, correct dosing and optimal method of delivery.

## Supporting Information

S1 Fig β2GPI deposition in cardiac tissue after cardiac IRI. Representative images of (A) A mouse subjected to 30 min ischemia and 24 h reperfusion with no β2GPI deposition apparent outside the AAR; (B) β2GPI evident within the AAR in the same mouse. (Marker = 20 μm; Blue = nuclear staining DAPI, green = β2GPI).
(PDF)
