# Supplementary material for: The Fifth Domain of Beta 2 Glycoprotein I Protects from Natural IgM Mediated Cardiac Ischaemia Reperfusion Injury
Source: PLoS One. 2016 Mar 31;11(3):e0152681. doi: 10.1371/journal.pone.0152681 (PMC4816326; doi:10.1371/journal.pone.0152681)
Supplement: S1 Fig — Representative images of (A) A mouse subjected to 30 min ischemia and 24 h reperfusion with no β2GPI deposition apparent outside the AAR; (B) β2GPI evident within the AAR in the same mouse. (Marker = 20 μm; Blue = nuclear staining DAPI, green = β2GPI). (PDF) [file pone.0152681.s001.pdf]

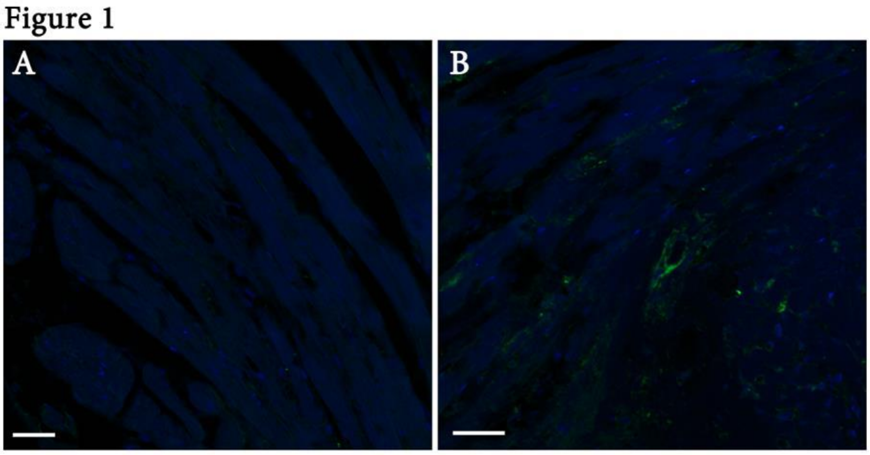

S1 Figure: **β2GPI deposition in cardiac tissue after cardiac IRI.**

Representative images of (A) A mouse subjected to 30 min ischemia and 24 h reperfusion with no β2GPI deposition apparent outside the AAR; (B) β2GPI evident within the AAR in the same mouse. (Marker = 20μm; Blue = nuclear staining DAPI, green = β2GPI).
